# Suppression of adenine nucleotide translocase-2 by vector-based siRNA in human breast cancer cells induces apoptosis and inhibits tumor growth *in vitro *and *in vivo*

**DOI:** 10.1186/bcr1857

**Published:** 2008-02-12

**Authors:** Ji-Young Jang, Yun Choi, Yoon-Kyung Jeon, Chul-Woo Kim

**Affiliations:** 1 Department of Pathology, Tumor Immunity Medical Research Center, Cancer Research Institute, Seoul National University College of Medicine, 28 Yongon-dong, Jongno-gu, Seoul 110-799, South Korea

## Abstract

**Introduction:**

Adenine nucleotide translocator (ANT) 2 is highly expressed in proliferative cells, and ANT2 induction in cancer cells is known to be directly associated with glycolytic metabolisms and carcinogenesis. In addition, ANT2 repression results in the growth arrest of human cells, implying that ANT2 is a candidate for cancer therapy based on molecular targeting.

**Methods:**

We utilized an ANT2-specific RNA interference approach to inhibit ANT2 expression for evaluating its antitumor effect *in vitro *and *in vivo*. Specifically, to investigate the therapeutic potential of ANT2 repression, we used a DNA vector-based RNA interference approach by expressing shRNA to knockdown ANT2 in breast cancer cell lines overexpressing ANT2.

**Results:**

ANT2 shRNA treatment in breast cancer cell line MDA-MB-231 repressed cell growth as well as proliferation. In addition, cell cycle arrest, ATP depletion and apoptotic cell death characterized by the potential disruption of mitochondrial membrane were observed from the ANT2 shRNA-treated breast cancer cells. Apoptotic breast cancer cells transfected with ANT2 shRNA also induced a cytotoxic bystander effect that generates necrotic cell death to the neighboring cells. The intracellular levels of TNFα and TNF-receptor I were increased in ANT2 shRNA transfected cells and the bystander effect was partly blocked by anti-TNFα antibody. Ultimately, ANT2 shRNA effectively inhibited tumor growth *in vivo*.

**Conclusion:**

These results suggest that vector-based ANT2 RNA interference could be an efficient molecular therapeutic method for breast cancer with high expression of ANT2.

## Introduction

Apoptosis can occur via a death receptor-mediated pathway or a mitochondrial pathway, and mitochondria-mediated apoptosis is initiated by multiple stimuli such as TNF, CD95 and stresses [1]. After receiving apoptotic signals, mitochondrial membrane permeability increases and the mediators such as cytochrome c and apoptosis-inducing factors are released to the cytoplasm, rapidly followed by the activations of caspase 9 and executive caspase 3 [2]. In healthy cells, mitochondrial membrane permeability is tightly controlled by voltage-dependent anion channels that are regulated by the interactions between Bcl2 family proteins [3,4].

Adenine nucleotide translocase (ANT) is a nuclear-encoded protein abundantly located in the inner mitochondrial membrane, and the role of this protein is to catalyze the exchange of mitochondrial ATP with cytosolic ADP. ANT therefore plays an important role in cellular energy metabolism by influencing mitochondrial oxidative phosphorylation. In addition, ANT is the major component of mitochondrial permeability–transition pore complex (PTPC) that interacts with Bcl2 family proteins, thereby contributing to mitochondria-mediated apoptosis [4,5]. ANT-deficient mice are able to form mitochondrial PTPC [6], however, inducing the argument about the roles of ANT in mitochondrial PTPC.

Human ANT has four isoforms (ANT1, ANT2, ANT3 and ANT4) and the relative expressions of these isoforms are dependent on developmental stages, proliferation status as well as tissue types or cell types. ANT3 is ubiquitously expressed in all tissues and the degrees of ANT3 expression are correlated with the levels of oxidative metabolism. ANT1 is highly expressed in terminally differentiated tissues such as skeletal muscles, heart and brain, whereas ANT4 is a murine stem and germ cell-specific isoform whose DNA methylation plays a key role in its transcriptional silencing in somatic cells [7,8]. On the other hand, ANT2 is specifically expressed in undifferentiated cells or tissues that are able to proliferate and regenerate; for example, the lymphocytes, kidney and liver [9-11]. The expression of ANT2 was recently found to be upregulated in several hormone-dependent cancers [12], and the induction of ANT2 expression in cancer cells was directly associated with glycolytic metabolisms, raising a question regarding the role of ANT2 during carcinogenesis [13-16]. Indeed, the overexpressions of ANT1 or ANT3 induce apoptosis while ANT2 lacks this proapoptotic activity [17,18]. ANT2 repression also leads to cell growth arrest and increases mitochondrial membrane potential from human cells as well as chemosensitized cancer cells [10,12], implying that ANT2 inhibits mitochondrial membrane permeability and acts as an antiapoptotic oncoprotein. We therefore hypothesized that ANT2 can be a promising candidate for cancer therapy based on specific molecular targeting.

iRNA is currently used for knockdown of a particular gene expression to identify the functions of a targeted gene and to examine the potential usage of a targeted gene as a therapeutic method. iRNA is an evolutionarily conserved phenomenon, and a multistep process produces active siRNA from RNase III endonuclease (Dicer). The consequent 21-nucleotide to 23-nucleotide siRNAs then mediate the degradation of complementary homologous RNA [19-21]. Two basic methods have currently been developed to selectively inhibit gene expression: the cytoplasmic delivery of short dsRNA oligonucleotides (siRNAs) that mimic the active intermediates of endogenous iRNA; and the nuclear delivery of gene expression cassettes encoding shRNAs that imitate micro iRNAs representing the active intermediates of different endogenous iRNA mechanisms. The mechanisms of DNA vector-based approaches involve the synthesis of small RNA from a DNA template under the control of RNA polymerase III (Pol III) promoter in transfected cells. Pol III has an advantage for directing the synthesis of small noncoding transcripts whose 3' ends are defined by the termination within a stretch of four to five thymidines [22-25]. These properties allow us to use DNA templates for generating small RNAs *in vivo *[26] whose structural features are close to the active siRNAs synthesized *in vitro*. The DNA vector-based iRNA approach can therefore be used widely for analyzing gene functions *in vitro *as well as *in vivo *[27-29].

In the present article we investigate the anticancer effects of ANT2 RNA interference in breast cancer models *in vitro *and *in vivo *using a DNA vector-based (H1-driven shRNA) iRNA approach. Our results demonstrate that the silencing of ANT2 expression using a DNA vector-based iRNA approach induces apoptotic cell death and cytotoxic bystander effects that elicit anticancer activity *in vitro *as well as tumor regression *in vivo*, implicating that the repression of ANT2 based on shRNA can be a novel method for breast cancer therapy.

## Materials and methods

### Cell lines and culture

The human metastatic breast carcinoma cell lines MCF7 and MDA-MB-231 as well as ovarian cancer cell lines SK-OV-3 and SNU8 were used throughout the study. These cells were provided by the Korean Cell Line Bank, Seoul, Korea and were cultured in DMEM supplemented with 10% FBS, 100 u/ml penicillin and 100 μg/ml streptomycin. MCF-10A, a line of healthy epithelial cells from human mammary gland (CRL-10317) provided by the American Type Culture Collection (Manassas, VA, USA) was used as a control. This cell line was maintained in the DMEM/Ham's Nutrient Mixture F-12 (1:1) with the addition of epidermal growth factor (20 ng/ml), cholera enterotoxin (100 ng/ml), insulin (10 μg/ml) and hydrocortisone (500 ng/ml) in the presence of 5% horse serum.

### Construction of the ANT2 siRNA expression vector

ANT2 siRNA-1, siRNA-2 and siRNA-3 were synthesized by Bioneer (Daejeon, Korea), and pSilencer™ 3.1-H1 puro plasmids for DNA vector-based siRNA synthesis were purchased from Ambion (Austin, TX, US). The oligonucleotide pairs of ANT2 siRNA-1, siRNA-2, and siRNA-3 are complementary to exon 2 or exon 4 (Genbank accession number NM001152), and the sequences of ANT2 siRNA-1, siRNA-2 and siRNA-3 are 5'-GCAGAUCACUGCAGAUAAGTT-3', 5'-CTGACATCATGTACACAGG-3' and 5'-GATTGCTCGTGATGAAGGA-3', respectively. The oligonucleotide pairs were designed to contain a terminal *BamH*I or *Hind*III restriction site for subcloning into the *BamH*I or *Hind*III site of pSilencer™ 3.1-H1 puro vector to generate pSilencer™ 3.1-H1 puro ANT2 siRNA vectors (shRNAs). These vectors produce a shRNA with a TTCAAGAGA linker sequence that forms looped structures. This linker is processed with Dicer to generate an ANT2-specific siRNA. A negative scrambled siRNA (Ambion) control with no significant homology to mouse or human gene sequences was designed to detect nonspecific effects.

### Transfection

For transfection, cells were plated on either six-well plates (2 × 10^5 ^cells per well) or 100 mm dishes (2 × 10^6 ^cells) and were allowed to adhere for 24 hours. Lipofectamine 2000 (Invitrogen, Carlsbad, CA, USA) was used for the transfections. pSilencer™ 3.1-H1 puro ANT2 siRNA vectors or pSilencer™ 3.1-H1 puro scramble siRNA vector were transfected into the cells. Transfected cells were then cultured for 4 hours and the culture media were replaced with fresh media supplemented with 10% FBS. The cells were harvested at 24–48 hours after transfection.

### Reverse transcription-polymerase chain reaction

After 48 hours of transfection, the cells were collected and total RNA was extracted using Trizol (Invitrogen) according to the manufacturer's instructions. For RT-PCR analysis, 5 μg total RNA was reverse-transcribed using RT-PCR kits (Promega, Madison, WI, USA). PCR was used to amplify target cDNA with the following conditions: 35 cycles of 94°C for 1 minute, 55°C for 1 minute and 72°C for 2 minutes. The PCR products were analyzed using standard agarose gel electrophoresis.

The primers used for RT-PCR are ANT1 forward, 5'-ACAGATTGTGTGGTTT-3' and reverse, 5'-TTTTGTGCATTAAGTGGTCTTT-3' ; ANT2 forward, 5'-CCGCAGCGCCGGAGTCAAA-3' and reverse, 5'-AGTCTGTCAAGAATGCTCAA-3' ; ANT3 forward, 5'-AACCAAGAGAACCACGTAGAA-3' and reverse, 5'-CTTAGAACAGACTTGGCTC-3' ; TNF-receptor I forward, 5'-CTGCCTCAGCTGCTCCAAA-3' and reverse, 5'-CGGTCCACTGTGCAAGAAGAG-3' ; and β-actin forward, 5'-GGAAATCGTGCGTGACATTAAGG-3' and reverse, 5'-GGCTTTTAGGATGGCAAG GGA C-3'.

### Western blotting

Anti-ANT, anti-Bcl-xL, anti-Bax, anti-α-tubulin as well as anti-caspase-3 antibodies were obtained from Santa Cruz Biotech (Santa Cruz, CA, USA) and polyclonal anti-ANT3 antibody was donated by Dr HH Schmid (University of Minnesota, MN, USA).

For western blot analyses, cells were harvested after 48 hours of transfection and were lysed with lysis buffer (5 mM/l ethylenediamine tetraacetic acid; 300 mM/l NaCl; 0.1% NP-40; 0.5 mM/l NaF; 0.5 mM/l Na_3_VO_4_; 0.5 mM/l phenylmethylsulfonyl fluoride; and 10 μg/ml each of aprotinin, pepstatin and leupeptin; Sigma, St Louis, MO, USA). After centrifugation at 15,000 × *g *for 30 minutes, the concentrations of supernatant proteins were analyzed by Bradford reagent (Bio-Rad, Hercules, CA, USA).

For the analysis of protein contents, 50 μg total proteins was electrophoresed in 10% SDS-PAGE gel, transferred to polyvinylidene difluoride membranes (Millipore, Bedford, MA, USA) and were then incubated with the respective antibodies indicated above. Immunoblots were visualized using an enhanced chemiluminescence detection system (Amersham Pharmacia Biotech, Uppsala, Sweden).

### Apoptosis and DNA fragmentation assays

Approximately 2 × 10^5^/ml MDA-MB-231 cells were transfected with respective pSilencer™ 3.1-H1 puro ANT2 siRNA-1, siRNA-2 and siRNA-3 vectors as well as with pSilencer™ 3.1-H1 puro scramble siRNA vector for the indicated times. The transfected cells were harvested, washed twice with PBS and were then incubated for 15 minutes at room temperature with a solution of annexin V conjugated with fluorescence isothiocyanate (2.5 μg/ml) and propidium iodide (PI) (5 μg/ml) (BD Pharmingen, San Diego, CA, USA) for flow cytometry (Epics XL; Coulter, Marseille, France) to detect the levels of apoptosis. Genomic DNA was extracted using genomic DNA extraction kits (G-DEX™IIc; Invitrogen, Seoul, Korea) and was subjected to electrophoresis in 2% agarose gels for DNA fragmentation analysis.

### ATP assay

ATP assays were conducted using CellTiter-Glo™ Luminescent Cell Viability assay kits (Promega) that quantify ATP levels in viable cells. This bioluminescence assay utilizes luciferase, which induces light emission during the interaction between ATP and luciferin. Lyophilized enzyme/substrate mixtures (250 μl) were transferred to opaque 96-well microplates containing cell lysates. The plates were incubated at room temperature for 10 minutes to stabilize luminescence signals and then the stabilized signals were quantified with an Orion Luminometer (Berthold Detection Systems, Oak Ridge, TN, USA).

### Cell cycle analysis

The cells transfected with pSilencer™ 3.1-H1 puro ANT2 siRNA or pSilencer™ 3.1-H1 puro scramble siRNA vector were trypsinized, counted, centrifuged and fixed in ethanol for 3 hours. These cells were then washed twice in PBS and centrifuged. Pellets were resuspended with a solution containing RNase (0.02 mg/ml) (Sigma), incubated at 37°C for 30 minutes and were stained with PI (0.02 mg/ml) (Sigma). The cells were analyzed by flow cytometry (Epics XL; Coulter).

### Measurement of mitochondrial membrane potentials

To measure mitochondrial membrane potential disruption, the cells transfected with pSilencer™ 3.1-H1 puro ANT2 siRNA or pSilencer™ 3.1-H1 puro scramble siRNA vector were harvested, washed twice with PBS and were incubated with 20 nM 3,3'-diethyloxacarbocyanine (Molecular Probes, Eugene, OR, USA) for 15 minutes at 37°C. Mitochondrial membrane potential values were determined by flow cytometry (Epix XL; Coulter).

### *In vitro *bystander effect assays

MDA-MB-231 cells (1.5 × 10^3^) were cultured with the culture media of pSilencer™ 3.1-H1 puro ANT2 siRNA-1/siRNA-2/siRNA-3-transfected cells for 24 hours and were then harvested. Cell death was determined by annexin V–fluorescence isothiocyanate/PI staining followed by flow cytometry analysis (Epics XL; Coulter).

### Fluorescent-activated cell sorter analysis (intracellular and surface staining)

Respective pSilencer™ 3.1-H1 puro ANT2 siRNA-1, siRNA-2 and siRNA-3 vectors as well as pSilencer™ 3.1-H1 puro scramble siRNA vector were transfected into MDA-MB-231 cells for the indicated times. Six hours before harvesting, the cells were treated with brefeldin A (10 μg/ml), washed twice with PBS, fixed with 2% paraformaldehyde, permeabilized with buffer (1% BSA, 0.1% saponine, 0.1% sodium azide in PBS) and were then stained with phenylethylene-conjugated anti-TNFα, anti-IFNγ, anti-IL-12 as well as anti-mouse IgG antibodies for 1 hour at 4°C (BD Pharmingen). Surface staining was performed with phenylethylene-conjugated anti-TNF-receptor 1 as well as anti-mouse IgG antibodies and then analyzed by flow cytometry (Epics XL; Coulter).

### Antitumor effect of ANT2 iRNA *in vivo*

For tumor challenges, we established tumor models in 6-week-old to 8-week-old Balb/c nude mice by subcutaneously injecting 5 × 10^6 ^MDA-MB-231 cells into the right flanks. Treatments were started from 2 weeks after tumor inoculation when tumor volumes were <100 mm^3^. Intratumoral injections of PBS, pSilencer™ 3.1-H1 puro scramble siRNA vector (100 μg) or respective pSilencer™ 3.1-H1 puro ANT2 siRNA-1, siRNA-2 and siRNA-3 supplemented with Lipofectamine 2000 (200 μl) were performed three times per day for 5 days. Tumor sizes were measured using a caliper every week until 56 days after tumor challenges, and tumor volumes calculated using the formula: *m*_1_^2 ^× *m*_2 _× 0.5236 (where *m*_1 _represents the shortest axis and *m*_2 _the longest axis)

### *In situ *detection of apoptosis detection

*In situ *detection of apoptosis was performed using ApopTag Fluorescein (Intergenco, New York, USA). After the blocking of endogenous peroxidase with 3% hydrogen peroxide for 5 minutes, the sections were digested with proteinase K (20 μg/ml) for 15 minutes at room temperature and were then treated with bovine testicular hyaluronidase (0.5 mg/ml) for 30 minutes at 37°C. DNA was end-labeled with deoxynucleotidyl transferase-mediated dUTP nick end-labeling and was detected with peroxidase-conjugated antidigoxigenin antibody. The reactivity was visualized by the mixtures of diaminobenzidine and hydrogen peroxide.

### Statistical analysis

Data were analyzed using the Student's *t *test. *P *< 0.05 was considered statistically significant.

## Results

### ANT2 expression markedly upregulated in human breast cancer cell lines and efficiently suppressed by RNA interference

**Figure 1:**
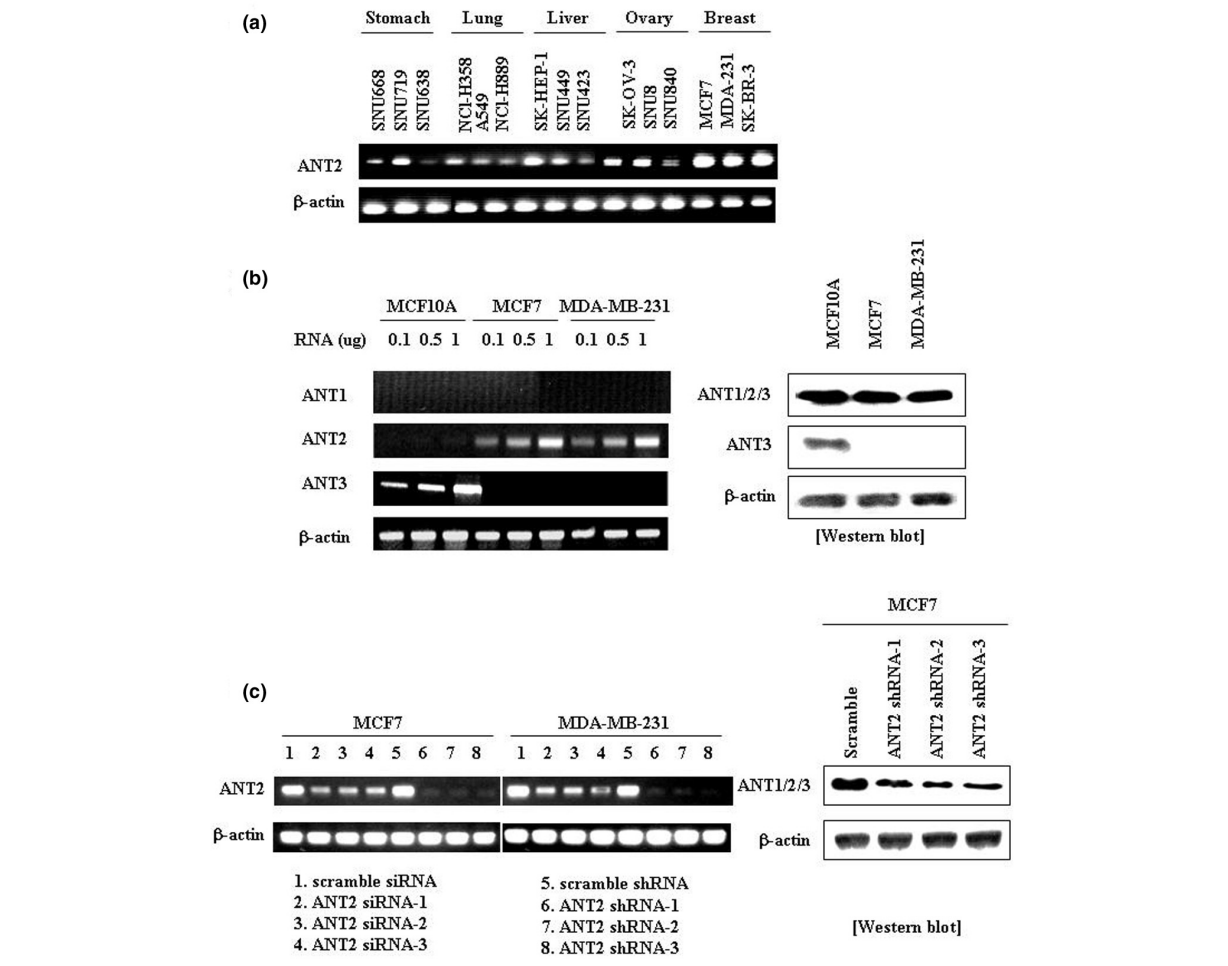
Expression and silencing efficiency of adenine nucleotide translocator 2 in cancer cell lines. Expression of adenine nucleotide translocator (ANT) 2 in cancer cell lines and the silencing efficiency of ANT2 siRNA and shRNA in human breast and ovarian cancer cell lines. **(a) **RT-PCR analysis for detecting ANT2 mRNA expression in various human cancer cell lines. To evaluate ANT2 expression levels in the human cancer cell lines of various origins (SNU668, SNU719, SNU638, NCI-H358, A549, NCI-H889, SK-HEP-1, SNU449, SNU423, SK-OV-3, SNU8, SNU840, MCF7, MDA-MB-231 and SK-BR-3), total RNA was extracted from respective cell lines and subjected to RT-PCR using specific primers for human ANT2 or β-actin (internal control). **(b) **RT-PCR analysis and western blotting to detect ANT isoform expressions in breast cancer cell lines as well as a non-neoplastic breast cell line. To compare ANT isoform mRNA levels in non-neoplastic breast epithelial cell line MCF10A with other breast cancer cell lines such as MCF7 and MDA-MB-231, total RNA was extracted from the respective cell lines and subjected to RT-PCR using specific primers for human ANT1/ANT2/ANT3 or β-actin. In addition, to detect ANT protein levels, total cell extracts were used for performing western blotting with anti-ANT, anti-ANT3 and anti-β-actin antibodies. **(c) **RT-PCR analysis and western blotting for detecting the level of ANT2 repression mediated by ANT2 siRNA and ANT2 shRNA. To assess the extinction of endogenous human ANT2 mRNA in MCF7 and MDA-MB-231 cells due to ANT2 RNA interference, respective cell lines were transfected with ANT2 siRNAs, ANT2 shRNAs, scramble siRNA as well as scramble shRNA for 48 hours. Total RNA was then extracted from respective samples and subjected to RT-PCR using specific primers for human ANT2 or β-actin. To indirectly detect the reduction of ANT2 protein by ANT2 RNA interference, MCF7 cells were transfected with ANT2 shRNA-1, shRNA-2 and shRNA-3 as well as scramble shRNA, and 48 hours later total cell extracts were collected for performing western blotting with anti-ANT and anti-β-actin antibodies.

To explore the functions of ANT2 in cancer, we first examined its mRNA expression levels in human cancer cell lines obtained from various organs (Figure 1a). RT-PCR showed that ANT2 mRNA was expressed in various human cancer cells originated from the stomach, lung, liver, ovary and breast. ANT2 mRNA was dramatically overexpressed in human breast cancer cell lines (MCF7, MDA-MB-231 and SK-BR-3) and was overtly increased in ovarian cancer cell lines (SK-OV-3 and SNU8), however, suggesting that overexpression of ANT2 could be a unique feature of breast cancer and ovarian cancer that may play a role in cancer development.

To expand the knowledge of ANT mRNA expressions among breast cancer cell lines, the MCF7 and MDA-MB-231 cell lines, as well as the non-neoplastic mammary epithelial cell line MCF10A, the relative expressions of three ANT isoforms from these cell lines were investigated at mRNA and protein levels (Figure 1b). In terms of mRNA expressions, ANT2 is highly expressed in both breast cancer cell lines while ANT1 and ANT3 expressions are not detectable. In contrast, the non-neoplastic MCF10A cell line barely expresses ANT2 as well as ANT1 but highly transcribes ANT3. Serendipitously, the transcriptional patterns of ANT isoforms in ovarian cancer cell line SK-OV-3 are identical to breast cancer cell lines (Additional File 1a), emphasizing the significance of ANT2 overexpression in breast cancer cells and ovarian cancer cells.

ANT2 protein levels were also tested from the breast cell lines by western blotting (Figure 1b). Owing to the absence of ANT1-specific and ANT2-specific antibodies, however, the protein levels of ANT2 were indirectly detected by subtracting the amount of ANT3 from total ANT protein levels. The total protein levels of ANT in respective breast cell lines are about the same, but ANT3 protein is not detectable from the breast cancer cell lines. On the other hand, ANT3 protein is dominantly expressed in the non-neoplastic MCF10A cell line where ANT3 mRNA is highly expressed. This result suggests that the breast cancer cell lines have more ANT1 and ANT2 proteins than ANT3 proteins. No ANT1 mRNA is detectable from both breast cancer cell lines, however, indicating the majority of ANT proteins in breast cancer cell lines might be ANT2 proteins. Collectively, these arrays of results suggest that the higher expression of ANT2 in breast cancer and ovarian cancer cell lines is a unique feature that may play a key role in cancer development.

Owing to the unique feature of overt ANT2 overexpression in breast cancer and ovarian cancer cell lines, we hypothesized that the downregulation of ANT2 in these cell lines may induce serious physiological effects that result in cell growth inhibition or cell death. To evaluate the functions of ANT2 in cancer development or the possible utility of ANT2 downregulation for breast cancer therapy, therefore, ANT2-specific knockdown experiments were performed with human breast cancer cell lines. Initially, respective siRNA-1, siRNA-2 and siRNA-3 oligonucleotides directed at exon 2 or exon 4 of ANT2 mRNA were synthesized to determine whether ANT2 siRNA suppresses ANT2 expression. At the same time, DNA oligonucleotides representing the siRNA duplex were cloned into pSilencer™ 3.1H1 puro vector to produce a high-level silencing effect based on a DNA vector system. The synthesized shRNA derived from DNA templates was composed of two identical 21-nucleotide sequence motifs in an inverted orientation, separated by a 9 base pair nonhomologous spacer (shRNA).

To confirm the silencing efficacies of ANT2 siRNA-1, siRNA-2 and siRNA-3 as well as pSilencer™ 3.1H1-ANT2 siRNA (ANT2 shRNA-1, siRNA-2 and siRNA-3), the cell lines MCF7 and MDA-MB-231 were transfected with the above samples as well as scramble siRNA and pSilencer™ 3.1H1-scramble siRNA (scramble shRNA) as negative controls. After culturing for 48 hours, their efficacies in extinguishing ANT2 mRNA expression were evaluated by RT-PCR. As shown in Figure 1c, the treatment with ANT2 shRNA was much more effective than the treatment with ANT2 siRNA in terms of downregulating the ANT2 mRNA level. Treatment with ANT2 shRNA for 48 hours decreased ANT2 mRNA expressions in MCF7 and MDA-MB-231 cells by over 90% compared with the cells transfected with control vectors, while ANT2 siRNA treatment resulted in markedly less reduction. The total protein levels of ANT were also downregulated by ANT2 shRNA, indirectly indicating that the knockdown of ANT2 reduces the protein levels of ANT2 (Figure 1c). These results suggest that ANT2 shRNA is superior to ANT2 siRNA for downregulating ANT2 mRNA as well as protein levels and can be used to target ANT2 for breast cancer therapy.

Taken together, we have uniquely identified the higher expression of ANT2 mRNA from human breast cancer cell lines MCF7, MDA-231 and SK-BR-3 as well as ovarian cancer cell lines SK-OV-3 and SNU8. To investigate the possible utility of ANT2 downregulation as a cancer therapy, ANT2 shRNA systems were adopted and the actual downregulation of ANT2 mRNA as well as protein mediated by ANT2 shRNA was confirmed from human breast cancer cell lines. As a result, we established a shRNA system to investigate the therapeutic value of ANT2 downregulation in breast cancer *in vitro *and *in vivo*.

### ANT2 depletion induces cell cycle arrest (G_1 _arrest) and apoptotic cell death *in vitro*

**Figure 2:**
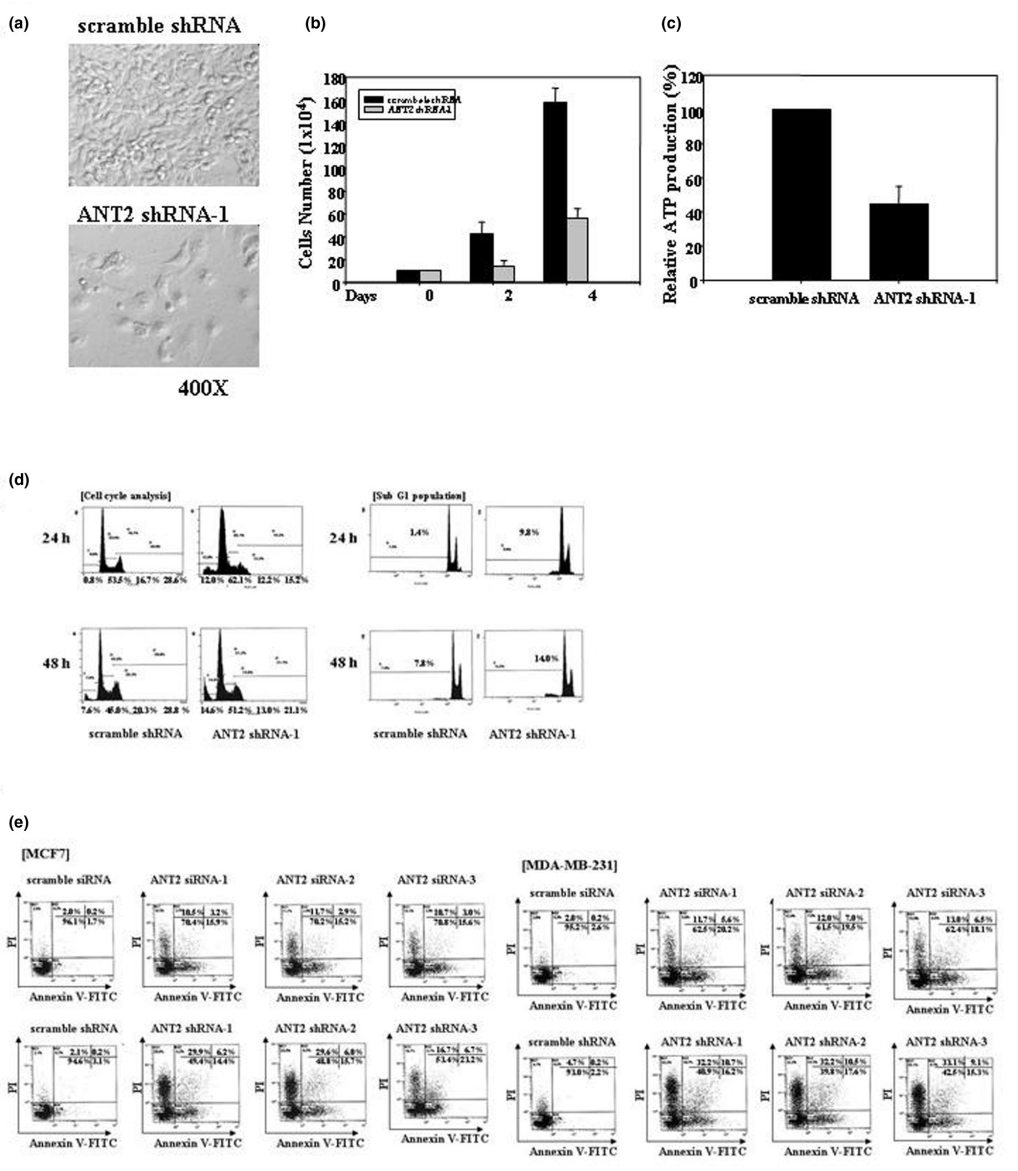
Apoptotic changes of human breast cancer cell lines transfected with adenine nucleotide translocator 2 shRNA. **(a) **Cell morphology analysis: MDA-MB-231 cells were transfected with scramble shRNA or adenine nucleotide translocator (ANT) 2 shRNA-1 and then examined under a phase contrast microscope after 24 hours of transfection. **(b) **Cell proliferation assay: after 2 or 4 days of transfection with ANT2 shRNA-1 as well as scramble shRNA, the numbers of viable cells were determined using a hemacytometer after staining dead cells with Trypan Blue. **(c) **ATP assay: cells were transfected with ANT2 shRNA-1 as well as scramble shRNA and then lysed to quantify total intracellular ATP levels after 24 hours of incubation. Results are tabulated in relative ATP production by normalizing luminescence units (RLU) with total protein levels. **(d) **Cell cycle analysis: after 24 or 48 hours of transfection with scramble shRNA and ANT2 shRNA-1, the cells were trypsinized, fixed in ethanol and stained with propidium iodide to determine DNA contents. Cell cycle distributions were analyzed by flow cytometry. **(e) **Apoptosis analysis: cells were transfected with specific siRNA or shRNA against ANT2, and then 48 hours later the transfected cells were stained with annexin V–fluorescence isothiocyanate (FITC) and propidium iodide (PI) for flow cytometry analysis. Data are representative of three independent experiments.

To explore the potential of ANT2 shRNA as a treatment for human breast cancer, the diverse phenotypic changes of respective cancer cell lines affected by ANT2 shRNA were investigated. Firstly, the cell survival and proliferation rate were investigated using MDA-MB-231. After transfecting MDA-MB-231 cells with ANT2 shRNA-1 for 24 hours, the transfected cells became less confluent as compared with the cells transfected with scramble shRNA. Many ANT2 shRNA transfected cells even became rounded and detached from culture plates, providing one line of evidence about cell death or cell cycle arrest (Figure 2a). In addition, the proliferation rate of ANT2 shRNA-1 transfected MDA-MB-231 cells was also obviously reduced (Figure 2b). To understand the effects of ANT2 shRNA in MDA-MB-231 cells more specifically, the levels of intracellular ATP and the cell cycle status in transfected cells were investigated. Intracellular ATP levels were significantly reduced by up to 50% in the cells transfected with ANT2 shRNA-1 (Figure 2c), suggesting the death of ANT2 shRNA transfected cells is ascribed to the reduction of ATP synthesis. Cell cycle analysis indicated that the G_1 _populations of ANT2 shRNA-treated MDA-MB-231 cells were 12% (24 hours) and 14.6% (48 hours), while scramble shRNA-treated cells displayed 0.8% (24 hours) and 7.6% (48 hours) of the G_1 _populations (Figure 2d). Additional analysis gating on the sub-G_1 _population also showed similar results after 24 or 48 hours of transfection, indicating ANT2 shRNA induces G_1 _arrest in breast cancer cells.

We finally tested whether the knockdown of ANT2 shRNA induces cell death in the breast cancer cell lines MCF7 and MDA-MB-231 *in vitro*. Based on annexin V and PI staining, both ANT2 siRNA and ANT2 shRNA increased early apoptotic cells (AV^+^PI^-^), intermediate apoptotic cells (AV^+^PI^+^) as well as late apoptotic cells (AV^-^PI^-^) compared with control groups after 48 hours of transfection (Figure 2e). ANT2 shRNA was more effective than ANT2 siRNA, however, in terms of inducing cell death. For example, ANT2 siRNA induced around 25–35% of cell death, while ANT2 shRNA generated about 50–60% of apoptotic cells. These results coincide with our previous observation regarding the higher efficacy to suppress ANT2 expression by ANT2 shRNA than ANT2 siRNA as shown in Figure 1c, suggesting increased ANT2 downregulation induces more cell death. Additionally, the ovarian cancer cell lines SK-OV-3 and SNU8 transfected with ANT2 siRNA or shRNA demonstrated similar results to those obtained from the breast cancer cell lines (Additional File 1b). These results imply that the physiological effects of ANT2 downregulation could be consistent in both cancers.

Taken together, these results suggest that the downregulation of ANT2 efficiently inhibits cell growth and proliferation by reducing ATP levels as well as inducing G_1 _arrest, which ultimately leads to the apoptosis of human breast cancer cells *in vitro*.

### ANT2 shRNA induces apoptosis by reducing the membrane potential of mitochondria and activating caspase 3 in MDA-MB-231 cells

**Figure 3:**
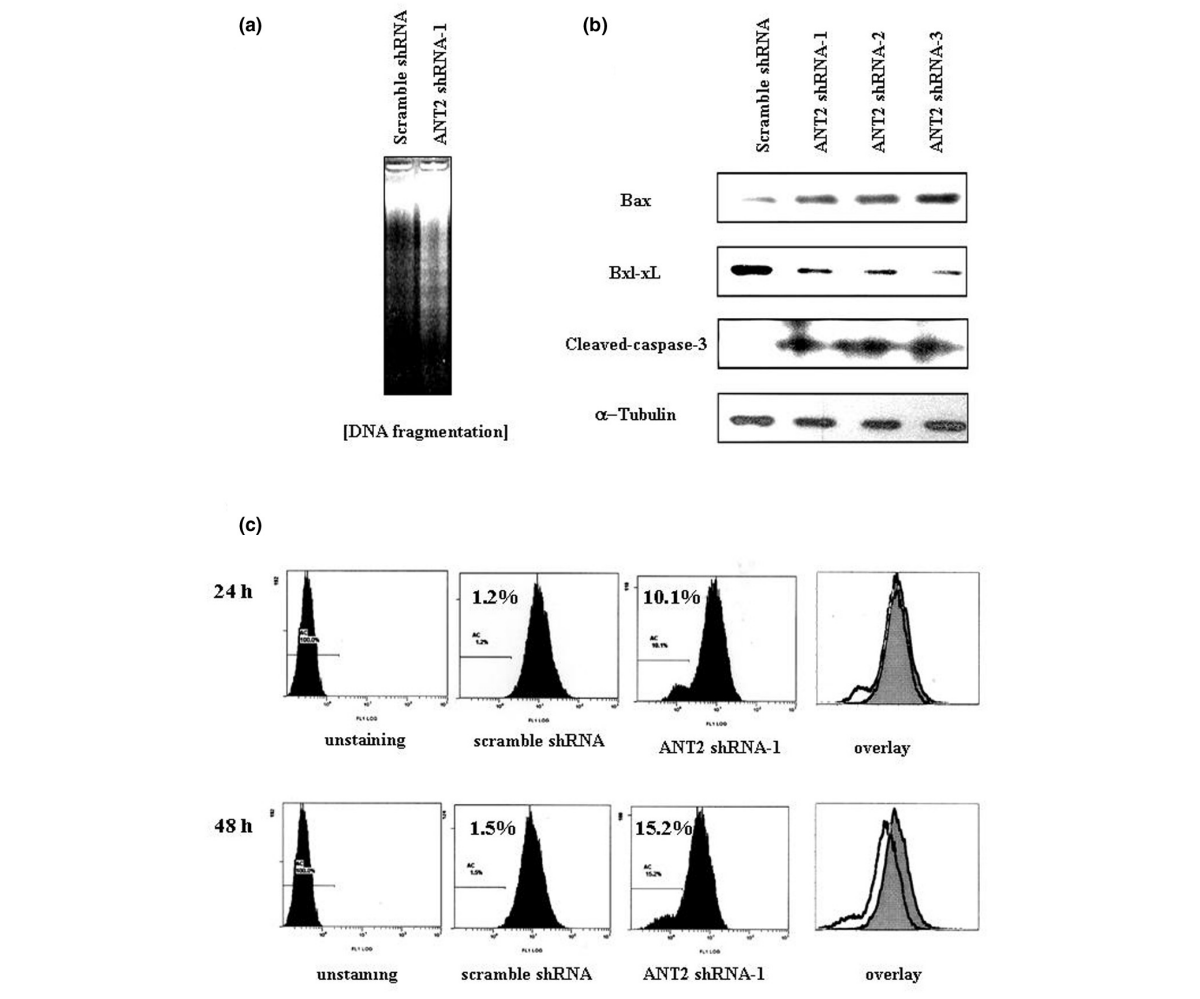
Adenine nucleotide translocator 2 shRNA regulated Bcl-2 family members and disrupted mitochondrial membrane potentials. Adenine nucleotide translocator (ANT) 2 shRNA regulated the levels of Bcl-2 family members and disrupted mitochondrial membrane potentials to induce apoptosis in MDA-MB-231 cells. **(a) **DNA laddering assay: cells were transfected with ANT2 shRNA-1, and then 48 hours later total genomic DNA was extracted and subjected to electrophoresis in 2% agarose gel to examine DNA fragmentation patterns. **(b) **Western blotting for identifying apoptotic mechanisms: cells were transfected with ANT2 shRNA-1, shRNA-2 and shRNA-3. Cytoplasmic extracts (free of mitochondria) were prepared after 24 hours of incubation for performing western blotting with anti-Bax, anti-Bcl-xL, anti-cleaved caspase 3 and anti-α-tubulin antibodies. **(c) **Analysis for detecting mitochondrial membrane potential: cells were transfected with ANT2 shRNA-1, and then 24 or 48 hours later the transfected cells were stained with 3,3'-diethyloxacarbocyanine. Mitochondrial membrane potentials were quantified by flow cytometry. Data are representative of three independent experiments.

To confirm apoptosis was responsible for the cell death induced by ANT2 shRNA in breast cancer cell lines, we examined the presence of genomic DNA fragmentation. As we expected, DNA laddering – a characteristic of apoptotic cell death – was abundantly observed from ANT2 shRNA-1 transfected MDA-MB-231 cells (Figure 3a), proving the death of transfected cells mediated by ANT2 shRNA is apoptotic.

It has been reported that Bcl-2 family molecules can physically interact with ANT and that this interaction regulates ANT-mediated mitochondrial permeability transition pore formation [7]. To understand the mechanisms of apoptosis induced by ANT2 shRNA, we examined the changes of Bcl-2 family protein levels during the ANT2 shRNA-1-induced, shRNA-2-induced and shRNA-3-induced apoptosis in MDA-MB-231 cells by western blotting. The transfection of ANT2 shRNA-1, shRNA-2 and shRNA-3 resulted in the upregulation of Bax (proapoptotic) and the downregulation of Bcl-xL (antiapoptotic) (Figure 3b). In addition, caspase-3 activation – defined by the appearance of cleaved caspase 3 – was observed from ANT2 shRNA-treated cells. These results clearly demonstrate that ANT2 shRNA induces the apoptosis via the Bcl-2 family.

We assumed the apoptosis triggered by ANT2 shRNA must be involved in the mitochondria-mediated apoptotic pathway because ANT2 protein is abundant in mitochondrial membranes. The potentials of mitochondrial membranes were measured by 3,3'-diethyloxacarbocyanine staining to prove our hypothesis. As expected, the cells transfected with ANT2 shRNA-1 displayed about 10-fold shifted curves to the left after 24 and 48 hours of transfection, indicating that mitochondrial membrane potentials are disrupted in apoptotic cells transfected with ANT2 shRNA (Figure 3c).

Taken together, these results implicate that ANT2 shRNA changes the Bcl-2 family balance in mitochondrial membranes, favoring a proapoptotic pore-forming status, and thereby causes the disruption of mitochondrial membrane potentials resulting in cell apoptosis.

### Cell death by ANT2 shRNA amplified by the cytotoxic bystander effect in MDA-MB-231 cells

Before transfecting MDA-MB-231 cells with ANT2 shRNA-1, shRNA-2 and shRNA-3, we also assessed transfection efficiencies using GFP (Green Fluorescence Protein)-expressing vector. Although the transfection efficiency was only about 30%, the proportion of cells killed by ANT2 shRNA treatment exceeded 60%, raising the possible existence of bystander effect. To confirm the presence of a bystander effect in MDA-MB-231 cells, we cultured nontransfected cells with the supernatants obtained from ANT2 shRNA-1, shRNA-2 and shRNA-3 transfected cells and then evaluated cell death by annexin V–PI staining.

**Figure 4:**
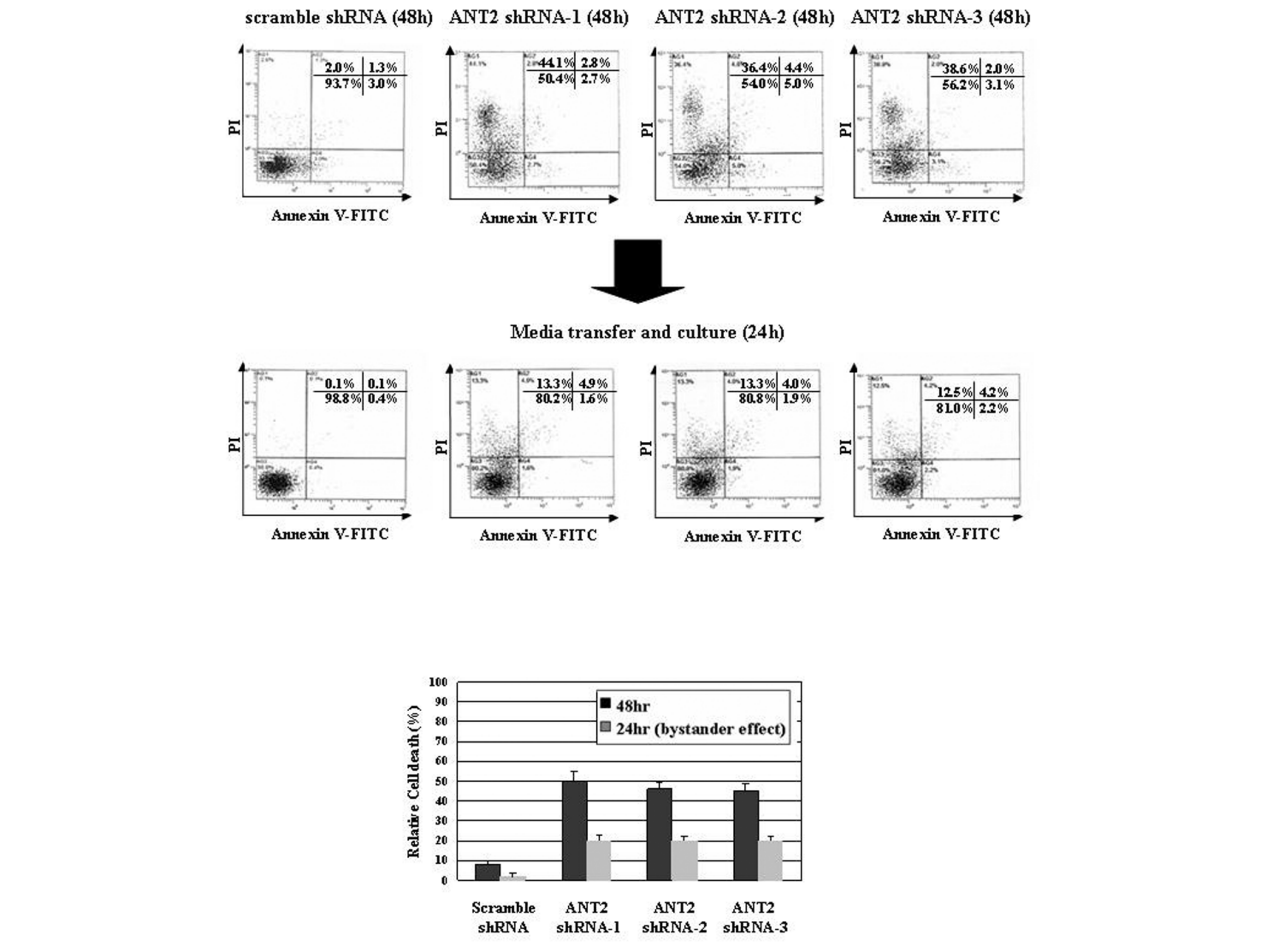
Adenine nucleotide translocator 2 shRNA induced cytotoxic bystander effects on MDA-MB-231 cells. To evaluate bystander effects, MDA-MB-231 cells were transfected with adenine nucleotide translocator (ANT) 2 shRNA-1, shRNA-2 and shRNA-3. After 48 hours of incubation, nontransfected cells were cultured with the media obtained from ANT2 shRNA-1, shRNA-2 or shRNA-3 transfected cells for the next 24 hours. These nontransfected cells were then stained with annexin V–fluorescence isothiocyanate (FITC) and propidium iodide (PI) for flow cytometric analysis. Data are representative of three independent experiments.

As shown in Figure 4, nontransfected cells cultured with the supernatants from ANT2 shRNA-1, shRNA-2 and shRNA-3 transfected cells committed to cell death defined by AV^-^PI^+^. The absence of DNA laddering and the lack of an AV^+ ^population lead us to consider that necrosis might be the major mechanism of cell death induced by this bystander effect. These results therefore suggest that cytotoxic agents must be secreted from ANT2 shRNA-transfected cells to the media.

### Bystander effect associated with TNFα production and increased TNF-receptor I expression in ANT2 shRNA transfected cells

**Figure 5:**
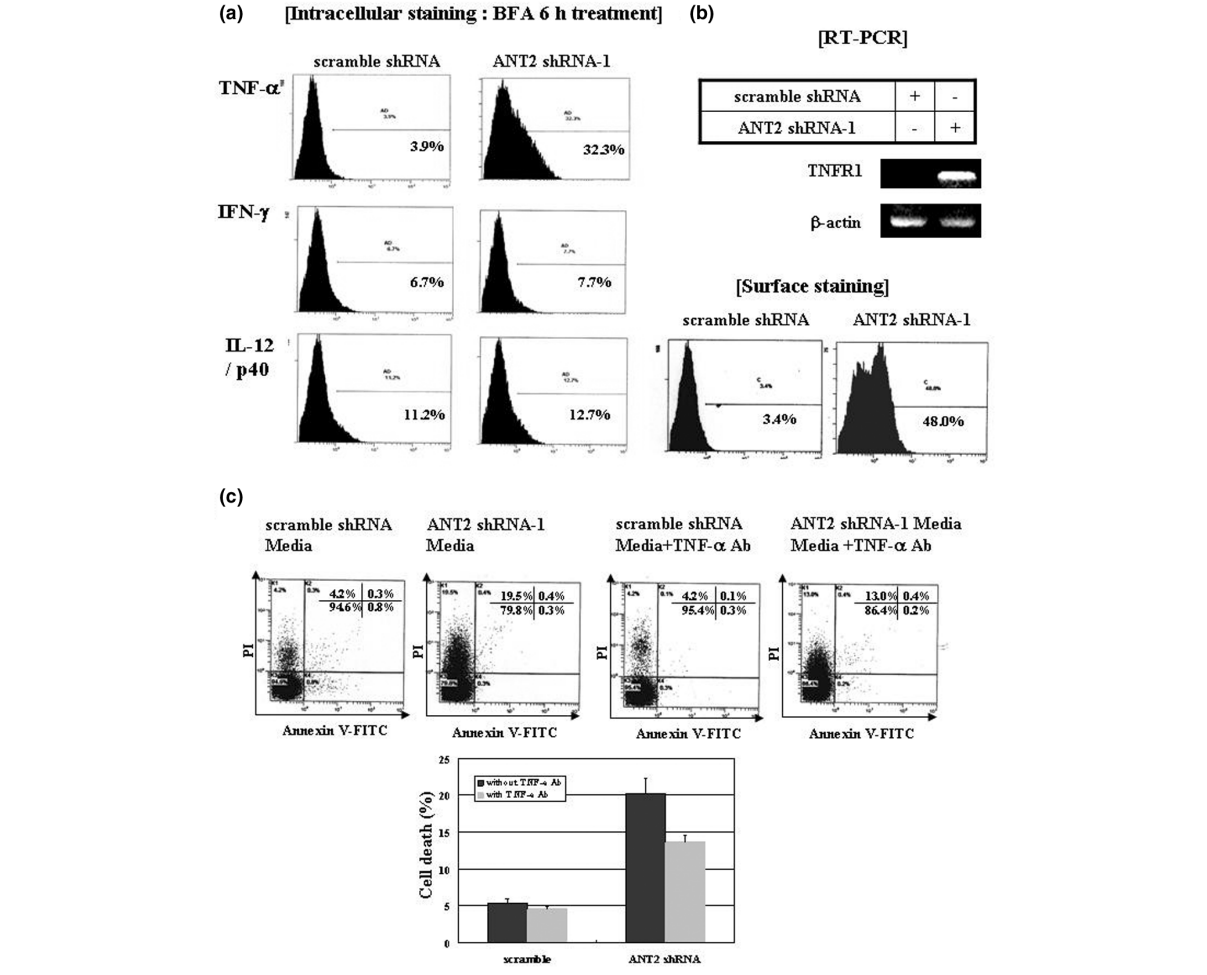
Bystander effects generated in MDA-MB-231 cells associated with TNFα production and TNF-receptor I expression. Bystander effects generated by adenine nucleotide translocator (ANT) 2 shRNA in MDA-MB-231 cells were associated with TNFα production and TNF-receptor I (TNFRI) expression. **(a) **Intracellular staining of TNFα, IFNγ and IL-12 p40. Cells were transfected with ANT2 shRNA-1. After 48 hours of incubation, the transfected cells were treated with brefeldin A for the next 6 hours. The cells were harvested, fixed in paraformaldehyde and then stained with phenylethylene-conjugated anti-TNFα, IFNγ or IL-12 p40 as well as anti-mouse IgG antibodies (negative control). Intracellular levels of TNFα, IFNγ and IL-12 p40 were analyzed by flow cytometry. **(b) **RT-PCR and fluorescent-activated cell sorter analysis for detecting the expression of TNFRI at transcriptional and translational levels. Cells were transfected with ANT2 shRNA-1. After 24 hours of incubation, total RNA was extracted and subjected to RT-PCR using specific primers for human TNFRI and β-actin. The RT-PCR products were analyzed by 1% agarose gel electrophoresis. In addition, the surface expression of TNFRI was measured by flow cytometry after staining cells with phenylethylene-conjugated anti-TNFRI antibody. **(c) **Partial neutralization of bystander effect mediated by anti-TNFα antibody. Cells were transfected with ANT2 shRNA-1. After 48 hours of incubation, the supernatants were collected and then mixed with or without TNFα antibody before transferring into nontransfected cells. These nontransfected cells were cultured for the next 24 hours and stained with annexin V–fluorescence isothiocyanate (FITC) and propidium iodide (PI) for flow cytometric analysis.

To determine the cause of this bystander effect, we examined the levels of proinflammatory cytokines such as TNFα, IFNγ and IL-12 from MDA-MB-231 cells treated with ANT2 shRNA or scramble shRNA by intracellular fluorescent-activated cell sorter. TNFα expression was elevated eight-fold in the cells treated with ANT2 shRNA-1 but the levels of IFNγ and IL-12 were unchanged (Figure 5a).

In addition, we evaluated TNF family death receptor I expression by RT-PCR and flow cytometry. Both transcription and surface expression of TNF-receptor I were upregulated by the transfection with ANT2 shRNA (Figure 5b). Additionally, the bystander effect was partially neutralized by anti-TNFα antibody (Figure 5c). These results suggest that the observed bystander effect might be caused by TNFα secretion and TNF-receptor I expression.

### ANT2 shRNA inhibits tumor growth *in vivo*

**Figure 6:**
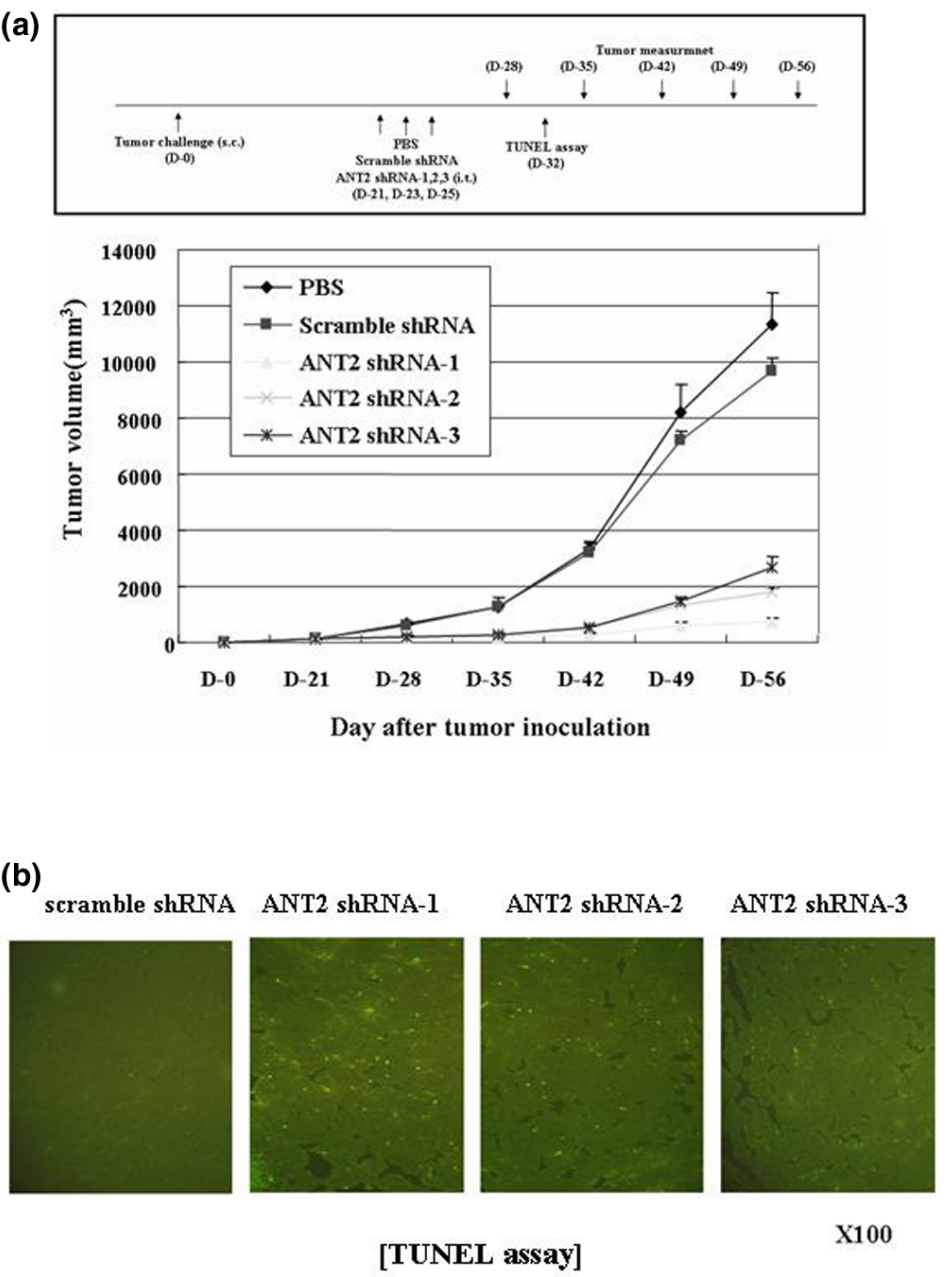
Adenine nucleotide translocator 2 shRNA inhibited tumor growth *in vivo*. **(a) ***In vivo *experimental schedule and tumor regression induced by adenine nucleotide translocator (ANT) 2 shRNA. Balb/c nude mice were challenged with 5 × 10^6 ^MDA-MB-231 cells by subcutaneous injection into the right flanks and then treated with PBS, scramble shRNA or respective ANT2 shRNA-1, shRNA-2 and shRNA-3 vectors supplemented with Lipofectamine 2000 by intratumoral injection on day 21, day 23 and day 25 post challenge. Tumor sizes were measured by a caliper every week and volumes calculated using *m*_1_^2 ^× *m*_2 _× 0.5236 (where *m*_1 _represents the short tumor axis and *m*_2 _the long axis) until day 56 post tumor challenge. **(b) **On day 32, tumor tissues were isolated and subjected to *in situ *apoptosis staining (Terminal deoxynucleotidyl transferase-mediated dUTP nick end-labeling (TUNEL) assay). Bright green dots represent apoptotic bodies.

We finally evaluated the antitumor effects of ANT2 shRNA *in vivo *using a nude mouse tumor xenograft model. Tumor sizes were measured twice per week and the observations lasted over 56 days after tumor challenge with MDA-MB-231 cell lines. Tumor growth was significantly inhibited in ANT2 shRNA-1-treated, shRNA-2-treated and shRNA-3-treated mice as compared with PBS-treated mice or scramble shRNA-treated mice (*P *< 0.05) (Figure 6a), suggesting intratumoral ANT2 shRNA treatment has a strong antitumor effect *in vivo*. One week after the final intratumoral injection of respective ANT2 shRNA-1, shRNA-2 and shRNA-3 (day 32), increased apoptotic cell death was detected by Terminal deoxynucleotidyl transferase-mediated dUTP nick end-labeling assay (Figure 6b) and the suppression of ANT2 was detected by RT-PCR (data not shown) from the tumor tissues obtained from ANT2 shRNA-treated mice. As a result, these data indicate that the injection of ANT2 shRNA induces tumor regressions associated with apoptosis *in vivo*.

## Discussion

Energy generation by cells in the form of ATP can be achieved in two main ways: oxidative phosphorylation in the mitochondria, and glycolysis in the cytoplasm. Most normal cells mainly utilize mitochondrial oxidative phosphorylation for ATP synthesis but switch to glycolysis at lower oxygen tension [30-34]. Cancer cells, however, typically depend upon glycolysis (the anaerobic breakdown of glucose to ATP) even in the presence of adequate oxygen, and this phenomenon is called the Warburg effect [35]. This increased glycolytic activity in cancer cells is suspected to possibly be due to mitochondrial defects and/or adaptation to a hypoxic tumor microenvironment [36-39]. The inhibition of glycolysis in cancer cells has therefore been suggested as a novel therapeutic strategy as well as a method to overcome drug resistances associated with mitochondrial dysfunctions [40,41].

ANT exchanges ADP and ATP across the mitochondrial inner membrane. Specifically, ANT1 and ANT3 export ATP produced by mitochondrial oxidative phosphorylation, whereas ANT2 imports ATP produced by glycolysis into mitochondria to supply the energy required for mitochondrial functions [14,16]. In fact, ANT2 is mainly expressed in proliferative and undifferentiated cells, and only trace amounts are existed in differentiated cells, suggesting ANT2 can be an anticancer therapeutic target. In addition to their role in energy metabolism, ANTs are also involved in the formation of mitochondrial PTPC that mediates the nonspecific release of apoptotic mediators. PTPC composed of ANTs, voltage-dependent anion channel, cyclophilin D and Bcl2-Bax family members is formed at the contact sites between mitochondrial inner and outer membranes [42,43]. In previous reports, the overexpression of ANT1 or ANT3 induced proapoptotic effects, whereas overexpression of ANT2 did not [17,18]. In addition, ANT2 suppression was also associated with cell growth arrest and the increase of mitochondrial permeability [9,11]. ANT2 is therefore considered an antiapoptotic oncoprotein that functions as an inhibitor of mitochondrial membrane permeability such as Bcl2 and Bcl-xL, supporting the idea to repress ANT2 as an anticancer strategy.

In the present study, we used DNA vector-based ANT2 siRNA as a tool to inhibit ANT2 transcripts. It was found that DNA vector-based ANT2 siRNA (shRNA) reduced ATP levels, induced cell cycle G_1 _arrest and effectively induced apoptotic cell death. Moreover, this apoptosis was accompanied by mitochondrial membrane potential disruption, cytochrome c release (data not shown) and caspase-3 activation. These facts encouraged us to speculate that ANT2 repression may disable ADP/ATP exchanges in mitochondrial membranes and/or promote PTPC formation as well as apoptosis. Interestingly, we also observed that ANT2 shRNA treatment upregulated proapoptotic Bax expression and downregulated antiapoptotic Bcl-xL expression. We speculate that these changes of gene expressions may contribute to the apoptotic cell death induced by ANT2 shRNA. Bcl2 family members in the mitochondrial membranes modulate the activities of ANTs, and the Bcl2 family and ANT interactively participate in the formation of PTPC [42,43]. No previous report, however, has demonstrated that ANTs can affect the expressions of Bcl2 family members. The specific mechanisms underlying this novel finding should be determined by further studies.

In contrast to our results, a recent study using siRNA oligonucleotides to knockdown ANT2 showed that ANT2 suppression neither induced apoptosis nor had any discernible effect on the mitochondrial network as well as the cell cycle. This suppression, however, did result in chemosensitization and increased mitochondrial transmembrane potential as well as reactive oxygen species levels [12]. These different observations between our report and others might be due to the utilization of different RNA interference technologies for suppressing ANT2 gene expression. Chemically synthesized siRNA is easily transfected into cancer cell lines and may be more effective in silencing targeted genes than shRNA vector [44]. siRNA synthesized *in vitro*, however, suppresses gene expression only for a short period, while shRNA expression vectors are transcribed and generated *in vivo *over long periods of time [45]. shRNA expression vectors therefore allow more sophisticated investigations and have been widely used for the functional analyses of genes and for anticancer therapies [28,29]. For example, when siRNA and shRNA iRNA technologies were utilized to knockdown the same gene, their effects were different [46,47]. In the present study, ANT2 shRNA was found to be more effective for suppressing ANT2 (Figure 1c) and induced more cell death than ANT2 siRNA (Figure 2e). As a result, our observations support that shRNAs synthesized from DNA templates can induce robust, lasting and near complete inhibitions of gene expression, suggesting shRNA is more applicable to gene therapy than siRNA [27].

Another important finding from the present study was the bystander effect induced by ANT2 shRNA treatment. We demonstrated that this phenomenon was due to TNFα secretion and TNF-receptor I upregulation from ANT2 shRNA-treated cells. In addition, the cell death induced by the bystander effect was mainly interpreted as necrosis. How the expressions of TNFα and TNF-receptor I were induced by ANT2 shRNA treatment, however, and why the bystander effect induced necrotic cell death, are not known. More experiments are needed to address these questions.

In a previous report, mitochondrial H^+^-ATPase inhibitor was found to increase mitochondrial membrane permeability, to decrease cellular ATP levels and to stimulate reactive oxygen species production, subsequently leading to the activation of stress-activated pathways and p21 induction [48]. In the present study using ANT2 shRNA, we observed the disruption of mitochondrial membrane potentials, ATP depletion, G_1 _stage arrest, apoptosis, the induction of TNFα and TNF-receptor I as well as the upregulation of surface and intracellular heat shock protein 70 expression *in vitro *(data not shown). Among the many common observations between two systems, the induction of heat shock proteins is notable from the immunological point of view. For example, the killing of tumor cells by a HSV-tk (herpes simplex virus thymidine kinase) gancyclovir system generated potent antitumor immunity and induced heat shock protein expression [49]. Interestingly, the induction of heat shock protein 70 expression induced the infiltrations of T cells, macrophages and predominantly dendritic cells into tumors, as well as the intratumoral expressions of T helper 1 cytokines such as IFNγ, TNFα and IL-12 [50,51]. We therefore speculate that ANT2 shRNA administration *in vivo *might accelerate tumor cell death and increase immune responses against tumors due to heat shock protein 70. A study is now underway to investigate the mechanisms of immune response associated with ANT2 shRNA in a wildtype mouse tumor xenograft model.

In summary, the knockdown of ANT2 using ANT2 shRNA systems in breast cancer models induces apoptotic cell death followed by ATP depletion, G_1 _stage arrest and the disruption of mitochondrial membrane potentials *in vitro *and tumor regression *in vivo*. In addition, the induction of TNFα and TNF-receptor I in breast cancer cells mediated by ANT2 shRNA generates the bystander effect that leads necrosis to the adjacent cells. We speculate that the antitumor effect mediated by ANT2 shRNA *in vivo *is mainly due to its apoptotic functions. The necrosis induced by the bystander effects, however, may contribute to the tumor regression *in vivo*. We are currently trying to evaluate the knockdown of ANT2 as a novel therapeutic model for breast cancer treatment based on our interesting observations.

## Conclusion

This present study demonstrates that ANT2 silencing by DNA vector-based iRNA effectively induces apoptotic cell death and tumor growth inhibition in breast cancer models *in vitro *and *in vivo*. We speculate that ANT2 silencing may offer a novel cancer therapeutic strategy for breast cancer.

## Abbreviations

ANT = adenine nucleotide translocator; BSA = bovine serum albumin; DMEM = Dulbecco's modified Eagle's medium; dsRNA = double-stranded RNA; FBS = fetal bovine serum; IFN = interferon; IL = interleukin; iRNA = interfering RNA; PBS = phosphate-buffered saline; PCR = polymerase chain reaction; PI = propidium iodide; PTPC = permeability–transition pore complex; RT = reverse transcription; shRNA = short-hairpin RNA; siRNA = small interfering RNA; TNF = tumor necrosis factor.

## Competing interests

The authors have applied for the domestic patent and will apply for the international patent regarding the utilization of ANT2 siRNA technology for a therapeutic method for cancer treatment. Seoul National University College of Medicine will retain the patent. The authors declare that they have no other competing interests.

## Authors' contributions

J-YJ performed most of the experiments and was responsible for producing the results, for the data analysis, and for preparing the manuscript. YC contributed to the conduction of additional experiments. Y-KJ was responsible for analysis of the data and for writing the paper. C-WK contributed to the design of the project, to data analysis and to writing of the paper. All authors reviewed and agreed the final manuscript.

## Supplementary Material

Additional file 1 Additional file 1 is a jpeg file containing images showing detection of apoptotic death of human ovarian cancer cell lines SK-OV-3 and SNU8 induced by adenine nucleotide translocator (ANT) 2 siRNA and shRNA. **(a) **Detection of ANT isoform mRNA from SK-OV-3. To evaluate ANT isoform mRNA in ovarian cancer cell line SK-OV-3, total RNA was extracted from this cell line and subjected to RT-PCR using specific primers for human ANT1/ANT2/ANT3 or β-actin. **(b) **Apoptosis analysis. Cells were transfected with specific siRNA or shRNA against ANT2, and then 48 hours later the transfected cells were stained with annexin V–fluorescence isothiocyanate (FITC) and propidium iodide (PI) for flow cytometry analysis. Data are representative of three independent experiments. Click here for file
