# Supplementary figures and images for: Suppression of adenine nucleotide translocase-2 by vector-based siRNA in human breast cancer cells induces apoptosis and inhibits tumor growth in vitro and in vivo
Source: Breast Cancer Res. 2008 Feb 12;10(1):R11. doi: 10.1186/bcr1857 (PMC2374967; doi:10.1186/bcr1857)

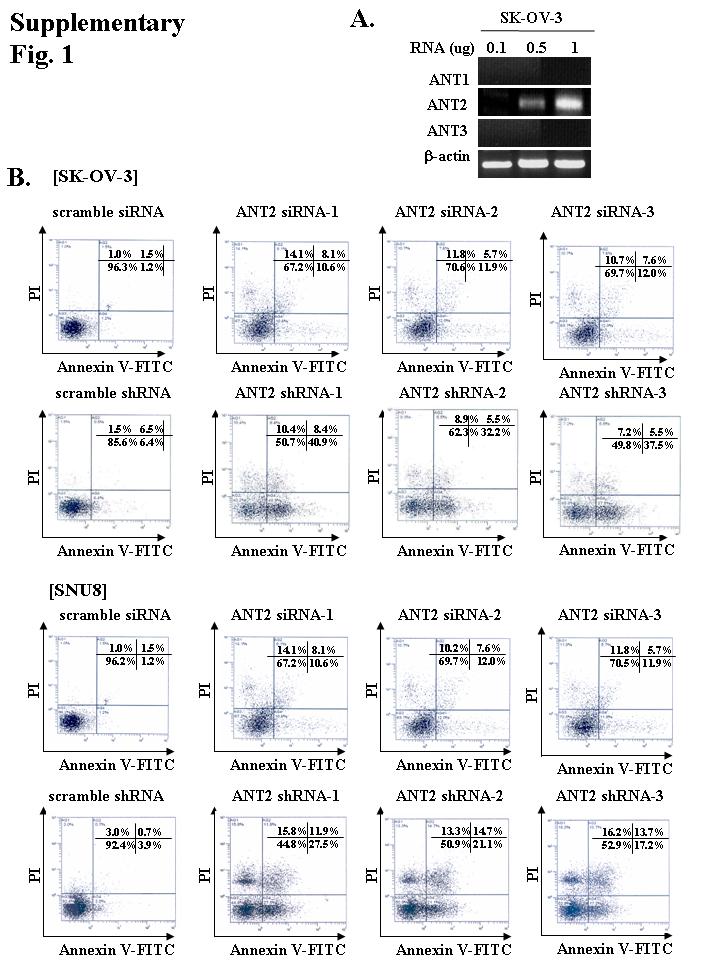

Supplement: Additional file 1 — is a jpeg file containing images showing detection of apoptotic death of human ovarian cancer cell lines SK-OV-3 and SNU8 induced by adenine nucleotide translocator (ANT) 2 siRNA and shRNA. (a) Detection of ANT isoform mRNA from SK-OV-3. To evaluate ANT isoform mRNA in ovarian cancer cell line SK-OV-3, total RNA was extracted from this cell line and subjected to RT-PCR using specific primers for human ANT1/ANT2/ANT3 or β-actin. (b) Apoptosis analysis. Cells were transfected with specific siRNA or shRNA against ANT2, and then 48 hours later the transfected cells were stained with annexin V–fluorescence isothiocyanate (FITC) and propidium iodide (PI) for flow cytometry analysis. Data are representative of three independent experiments. [file bcr1857-S1.jpeg]
